# The Impact of Host Diet on *Wolbachia* Titer in *Drosophila*

**DOI:** 10.1371/journal.ppat.1004777

**Published:** 2015-03-31

**Authors:** Laura R. Serbus, Pamela M. White, Jessica Pintado Silva, Amanda Rabe, Luis Teixeira, Roger Albertson, William Sullivan

**Affiliations:** 1 Department of Biological Sciences, Florida International University Modesto A. Maidique Campus, Miami, Florida, United States of America; 2 Biomolecular Sciences Institute, Florida International University Modesto A. Maidique Campus, Miami, Florida, United States of America; 3 Molecular, Cell, and Developmental Biology, University of California Santa Cruz, Santa Cruz, California, United States of America; 4 Instituto Gulbenkian de Ciência, Oeiras, Portugal; 5 Biology Department, Albion College, Albion, Michigan, United States of America; Monash University, AUSTRALIA

## Abstract

While a number of studies have identified host factors that influence endosymbiont titer, little is known concerning environmental influences on titer. Here we examined nutrient impact on maternally transmitted *Wolbachia* endosymbionts in *Drosophila*. We demonstrate that *Drosophila* reared on sucrose- and yeast-enriched diets exhibit increased and reduced *Wolbachia* titers in oogenesis, respectively. The yeast-induced *Wolbachia* depletion is mediated in large part by the somatic TOR and insulin signaling pathways. Disrupting TORC1 with the small molecule rapamycin dramatically increases oocyte *Wolbachia* titer, whereas hyper-activating somatic TORC1 suppresses oocyte titer. Furthermore, genetic ablation of insulin-producing cells located in the *Drosophila* brain abolished the yeast impact on oocyte titer. Exposure to yeast-enriched diets altered *Wolbachia* nucleoid morphology in oogenesis. Furthermore, dietary yeast increased somatic *Wolbachia* titer overall, though not in the central nervous system. These findings highlight the interactions between *Wolbachia* and germline cells as strongly nutrient-sensitive, and implicate conserved host signaling pathways by which nutrients influence *Wolbachia* titer.

## Author Summary

Many invertebrate organisms carry bacterial endosymbionts within their cells. In many cases, this ensures host access to resources provided by the endosymbionts, and reciprocally, a rich source of host-supplied nutrients supports bacterial growth and reproduction. However if bacterial reproduction is uncontrolled, an over-abundance of bacteria will ultimately destroy the host cell. Here we explore the factors that regulate endosymbiont abundance in host cells. We focused on *Wolbachia* endosymbionts that are carried naturally in the germ cells of fruit flies. Specifically, we determined whether dietary nutrients affect the amount of *Wolbachia* bacteria carried by female flies. We found that yeast-enriched diets strongly depleted *Wolbachia* in fly ovarian cells. By contrast, sucrose-enriched diets doubled the amount of *Wolbachia* in ovarian cells. In addition, we found that this response to diet is mediated through highly conserved TORC1 and insulin signaling pathways in the fly. Recent studies have revealed that host diet dramatically influences the types and abundance of gut microbes. Our study informs how host diet affects endosymbiotic bacteria housed within specific types of host cells.

## Introduction

Microbial endosymbionts have a profound impact on host metabolism and there are numerous examples in which microbes provide essential nutrients to the host [1–14]. In contrast, considerably less is known regarding how host metabolism and nutrition affect resident endosymbionts. To date, there is evidence that restricting the supply of host carbon, nitrogen and phosphorous significantly limits the number of *Chlorella* endosymbionts of green hydra and dinoflagellate endosymbionts of cnidarians [1]. Researchers have also observed that exposure to high levels of exogenous thiamine monophosphate suppresses the titer of *Sodalis* and *Wigglesworthia* endosymbionts in tsetse flies [15,16]. In this largely unexplored area, many outstanding questions remain: What are the host and endosymbiont metabolic and signaling pathways involved in nutrient sensing? To what extent do endosymbionts exhibit tissue-specific responses to nutrient availability? How are the rates of endosymbiont replication and cell death influenced by host metabolism and nutrients?

The symbiosis between *Wolbachia and Drosophila* is an excellent system to experimentally address these issues. *Wolbachia* are obligate intracellular endosymbionts carried by an estimated 40% of all insect species, including the established model organism *Drosophila melanogaster* [17–20]. Though *Wolbachia* endosymbionts are naturally carried within germline cells of both male and female insects, *Wolbachia* are ultimately removed from sperm prior to completion of spermatogenesis [17,18,21–25]. Thus, *Wolbachia* rely upon transmission through the maternal germline for their success. In addition to its functional importance in *Wolbachia* transmission, the well-characterized molecular and cell biology of *Drosophila* oogenesis has provided considerable contextual information and experimental tools that can be applied to studies of *Wolbachia*-host interactions [18,26–30].

The primary developmental units of the ovary that carry *Wolbachia* are referred to as egg chambers [27,28]. In each egg chamber, an outer layer of somatic follicle cells encapsulates an interconnected cyst of germline cells, comprised of 15 nurse cells and an oocyte. *Wolbachia* are initially loaded into these developing cysts during the first mitotic division from a *Wolbachia*-infected germline stem cell [18,31]. This germline *Wolbachia* population is amplified over time by binary fission and likely to some extent by exogenously invading *Wolbachia* [31–36]. *Wolbachia* persist in the germline throughout oogenesis, and a subset of the bacteria concentrate at the oocyte posterior pole during mid- to late oogenesis [31,37,38]. This ensures incorporation of *Wolbachia* into germline progenitor cells that form at the embryonic posterior pole, perpetuating the maternal germline transmission cycle [39]. Thus, maintenance of a sufficient *Wolbachia* titer in germline cells is important for success of the germline-based transmission strategy.

**Fig 1:**
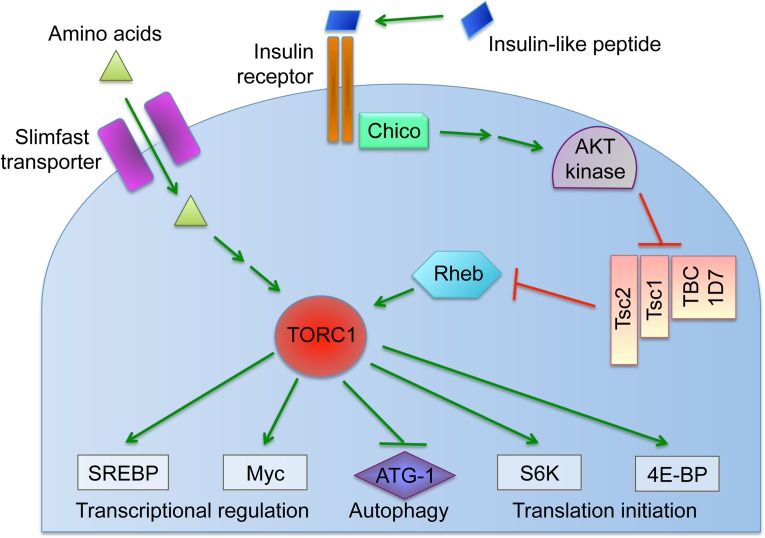
Overview of the nutrient-induced TORC1 signaling pathway.

Here we examined how host diet affects *Wolbachia* titer in *Drosophila melanogaster*. The data demonstrate that yeast-enriched diets suppress *Wolbachia* titer and lead to altered nucleoid morphology during oogenesis. Genetic and chemical disruptions indicate that the somatic insulin and TORC1 pathways (Fig. 1) are required for yeast-based suppression of oocyte *Wolbachia* titer. The data also indicate that sucrose-enriched diets increased oocyte *Wolbachia* titer, with little impact on nucleoid morphology. Evidence indicates that yeast-enriched diets substantially increase somatic *Wolbachia* titers, though this was not the case in the central nervous system (CNS). These studies demonstrate that *Wolbachia*, and likely other bacterial endosymbionts, exhibit distinct, tissue-specific responses to host nutrients that involve conserved signaling and metabolic pathways.

## Results

### Exposing *Drosophila* to a yeast-enriched diet suppresses germline *Wolbachia* titer

**Fig 2:**
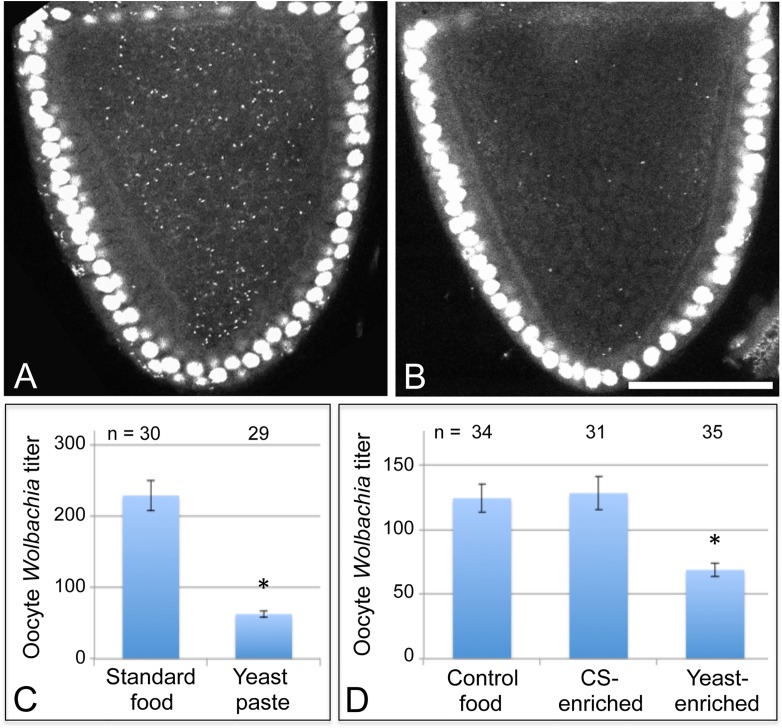
Host diet significantly impacts *Wolbachia* titer in *Drosophila* oogenesis. Stage 10A oocytes are outlined in red. Propidium iodide indicates *Drosophila* nuclei as large circles and *Wolbachia* as small puncta. A) *D*. *melanogaster* oocyte exposed to standard fly food. B) *D*. *melanogaster* oocyte exposed to yeast paste. Graphs indicate the average number of *Wolbachia* nucleoids within single focal planes of stage 10A oocytes. C) Oocyte *Wolbachia* titer comparison between control food and yeast paste conditions. D) *Wolbachia* titer response in *D*. *melanogaster* to 1:3 dilutions of water, corn syrup (CS), or yeast paste into standard food. Scale bar: 50 μm.

Nutrient availability strongly affects the life cycle of cultured bacteria, raising questions about how host nutrient conditions affect intracellular *Wolbachia* bacteria. As *D*. *melanogaster* in nature preferentially consume yeast [40–45], we tested the effect of dietary yeast on *Wolbachia* titer in vivo. Female flies were aged first for two days on standard food, then fed yeast paste for 3 days, and examined for *Wolbachia* titer in oogenesis. Ovarian tissues were stained with propidium iodide to label *Wolbachia* DNA, and the *Wolbachia* nucleoids imaged in oocytes of stage 10 egg chambers by confocal microscopy [38]. This analysis demonstrated that yeast paste-fed oocytes carried far less *Wolbachia* than control oocytes (Fig. 2A-B) (S1 Table). *Wolbachia* were further quantified within single oocyte focal planes to determine relative titer for each condition [32]. This revealed that *Wolbachia* titer in yeast paste-fed oocytes was at 27% of the control level. Oocytes treated with standard fly food exhibited an average of 229 +/- 21.1 *Wolbachia* puncta (n = 30), as compared to yeast paste-fed oocytes that carried 62.6 +/- 4.33 *Wolbachia* (n = 29) (p < 0.001) (Fig. 2C). This indicates that host exposure to yeast paste significantly reduces *Wolbachia* titer in oogenesis.

One possibility is that yeast paste diets reduce oocyte titer because other critical nutrients provided by standard fly food are unavailable. To address this issue, 2-day old *Drosophila* were fed with either standard food diluted 1/3 with water, thereafter referred to as “control food”, or fed with standard food diluted 1/3 with yeast paste, thereafter referred to as “yeast-enriched food” (S1 Table). After 3 days of exposure to these conditions, titer was assessed in oogenesis. The yeast-enriched condition exhibited 55% of the control titer level, with controls displaying 124 +/- 10.8 *Wolbachia* (n = 58), compared to yeast-enriched oocytes carrying 68.7 +/- 5.12 *Wolbachia* (n = 35) (p = 0.001) (Fig. 2D). To further assess whether this is due to differences in food hydration between control and yeast-enriched conditions, we also exposed flies to a 1/3 dilution of corn syrup into standard fly food (S1 Table). Although corn syrup-enriched food is less hydrated than control food, it resulted in similar oocyte titer measurements as the control, with an average of 128 +/- 12.9 *Wolbachia* visible per oocyte (n = 31) (Fig. 2D). These data together suggest that yeast-induced titer reduction is not due to depletion of specific nutrients or water available in standard food. Rather, the data indicate that dietary yeast is responsible for reducing *Wolbachia* titer carried by oocyte cells.

To determine whether dietary yeast can induce a similar oocyte titer response in wild insects as seen in laboratory fly stocks, *Drosophila melanogaster* and *Drosophila simulans* were collected from nature. These flies were exposed to yeast-enriched food and assessed for *Wolbachia* titer in oogenesis. We found that oocyte *Wolbachia* titer in the yeast-enriched condition was at 47% of the control level, with an average of 94.8 +/- 21.8 *Wolbachia* detected in control oocytes (n = 12), versus 44.6 +/- 6.52 *Wolbachia* detected in the yeast-enriched condition (n = 13) (p = 0.029) (S1 Fig). Thus, yeast-enriched diets suppress oocyte *Wolbachia* titer in wild-caught *Drosophila* analogous to laboratory *D*. *melanogaster* strains.

**Fig 3:**
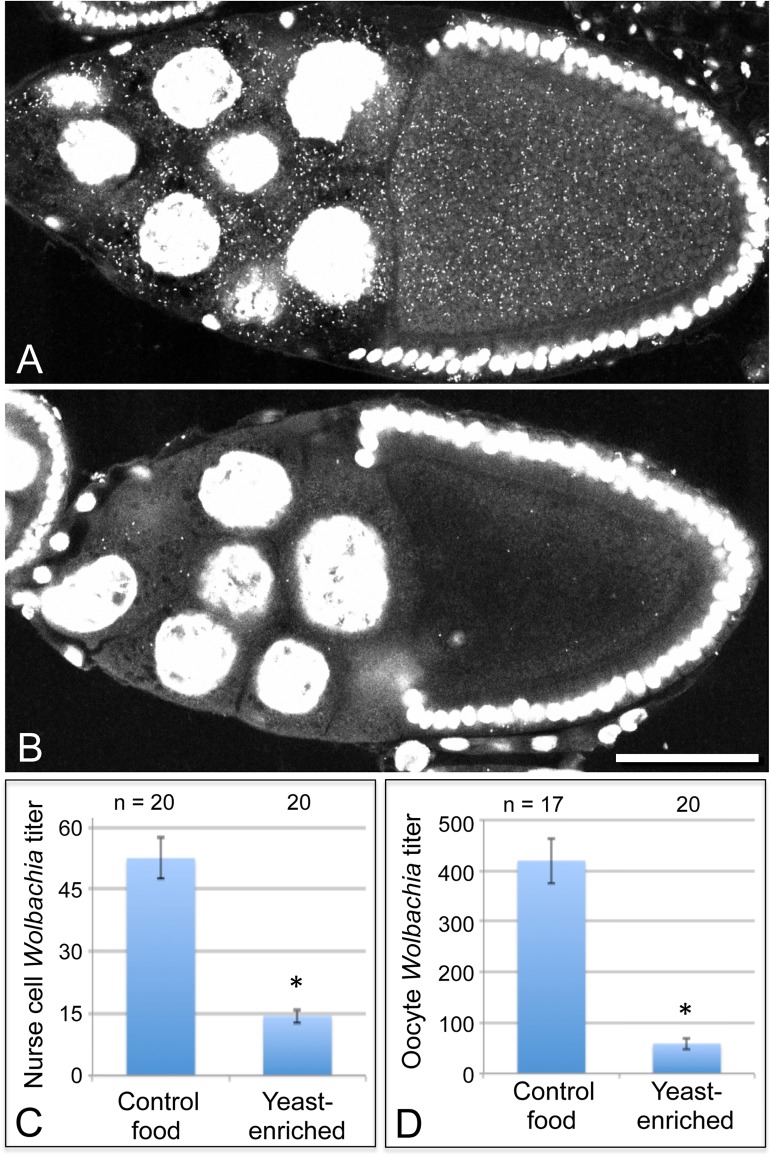
Dietary yeast affects *Wolbachia* titer in nurse cells as well as oocytes. Merged images show a full cross section from egg chambers raised on A) control food and B) yeast-enriched food. C-D) Average *Wolbachia* titer was determined for control vs. yeast-enriched conditions within a single egg chamber focal plane. C) Nurse cell titer values. D) Oocyte titer values from the same focal plane. Scale bar: 50 μm.

To further investigate the basis for yeast-associated *Wolbachia* depletion in oocytes, *Wolbachia* titer was examined in the germline-derived nurse cells associated with the oocyte. It is currently unclear in *Drosophila* when or how frequently *Wolbachia* travel through the ring canals between the nurse cells and oocyte. Thus, it is possible that *Wolbachia* depletion in oocytes could be due to preferential retention in the nurse cells. To investigate this, we imaged *Wolbachia* in equivalent focal planes of nurse cells and oocytes within single egg chambers and analyzed their *Wolbachia* titer [32]. Overlaid images showing a planar reconstruction of egg chambers indicated fewer *Wolbachia* throughout the germline cells of yeast-exposed organisms (Fig. 3A-B). Quantitation of the yeast-enriched condition indicated that nurse cells carried 27% of the control titer level (Fig. 3C). Specifically, 52.6 +/- 4.93 *Wolbachia* per nurse cell were detected in the control (n = 20), in contrast to 14.4 +/- 1.65 *Wolbachia* per nurse cell in the yeast-enriched condition (n = 20) (p < 0.001) (Fig. 3C). Furthermore, oocyte titer in the yeast-enriched condition was 14% of the control level, with 420 +/- 44.6 *Wolbachia* detected in control oocytes (n = 17), versus 59.0 +/- 11.1 *Wolbachia* in oocytes from the yeast-enriched condition (n = 20) (p < 0.001) (Fig. 3D). These data indicate that *Wolbachia* redistribution between germline cells is not responsible for the low oocyte titer observed in yeast-exposed organisms. Rather, yeast-enriched food induces similar *Wolbachia* depletion in nurse cells and oocytes.

### The nutrient-responsive kinase complex, TORC1, affects oocyte *Wolbachia* titer

**Fig 4:**
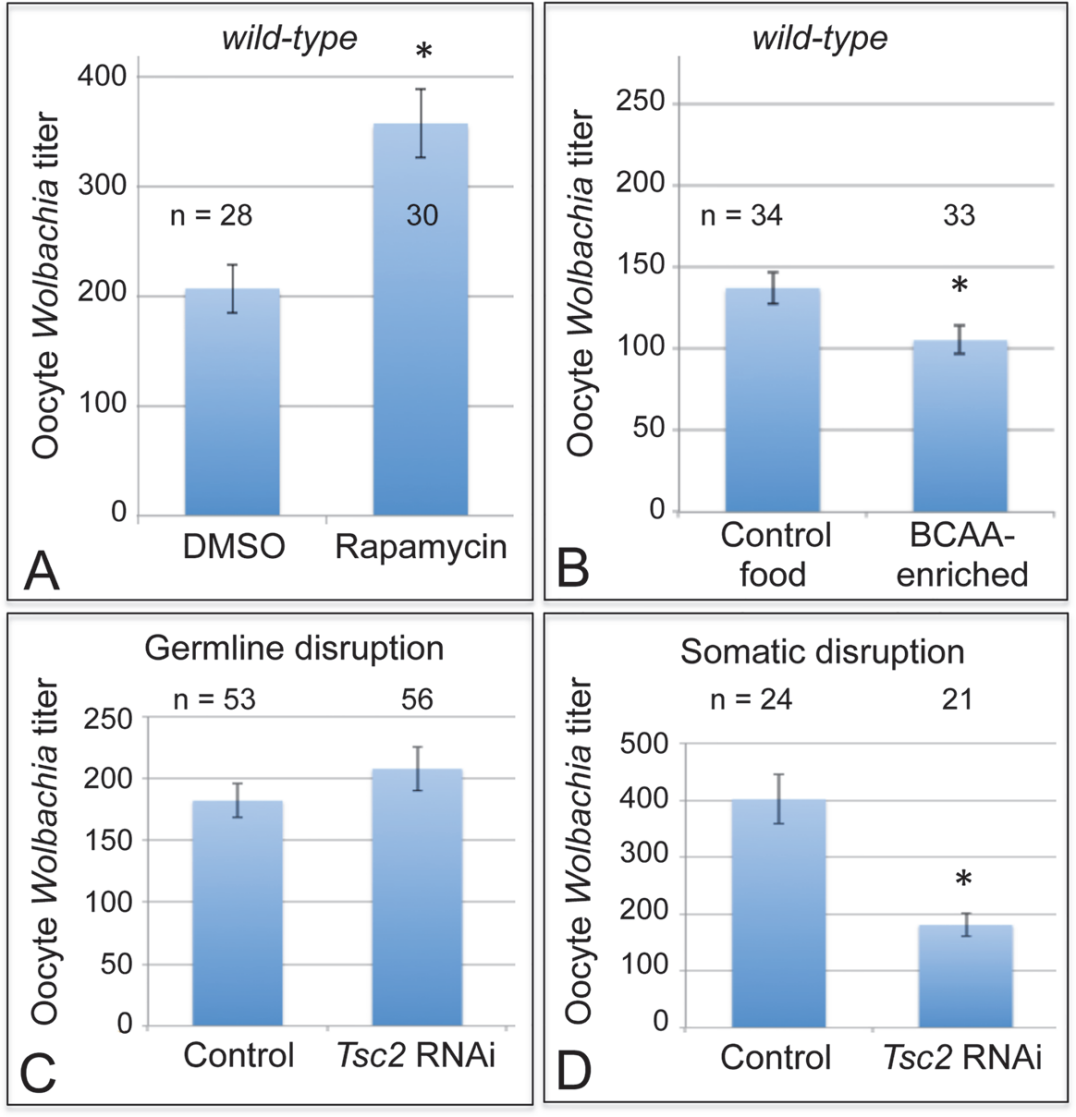
Somatic TORC1 activity affects *Wolbachia* titer in oogenesis. A) Average *Wolbachia* titer in oocytes treated with control DMSO or the mTORC1 inhibitor, Rapamycin. B) Titer was assessed in oocytes exposed to BCAA-enriched food. C-D) *Wolbachia* titer was also tested in flies carrying disruptions of the *Tsc2* gene, expected to elevate TORC1 activity. C) Genotypes used for germline *Tsc2* disruption: Control: *{nos-GAL4}/+; {nos-GAL4}/+*. *Tsc2* RNAi: *{nos-GAL4}/+; {nos-GAL4}/{UAS-Tsc2 dsRNA}*. D) Genotypes used for somatic *Tsc2* disruption: Control: *{da-GAL4}/+*. *Tsc2*: *{da-GAL4}/{UAS-Tsc2 dsRNA}*. * indicates a significant change in titer.

Cells coordinate intracellular events in response to exogenous nutrients using multiple signaling pathways that converge upon the Target of Rapamycin kinase complex 1 (TORC1) (Fig. 1) [46]. TORC1 can be activated by an amino-acid dependent signaling mechanism, or by insulin signaling (Fig. 1) [46–48]. To test whether TORC1 activity affects oocyte *Wolbachia* titer, flies were exposed to standard food containing the TORC1 inhibitor, rapamycin [49–52]. This experiment indicated that rapamycin treatment drove a 1.7-fold increase in oocyte *Wolbachia* titer (Fig. 4A). The average titer from control oocytes, exposed to DMSO-containing standard food, was 207 +/- 22.1 *Wolbachia* (n = 28). By contrast, oocytes exposed to rapamycin-containing standard food had 357 +/- 31 *Wolbachia* (n = 30) (p < 0.01) (Fig. 4A). Since rapamycin exposure leads to higher oocyte *Wolbachia* titer, this suggests that a normal consequence of TORC1 activity is suppression of oocyte *Wolbachia* titer.

If TORC1 function normally leads to decreased oocyte *Wolbachia* titer, then hyper-activation of TORC1 would be expected to drive a further reduction of oocyte titer. Branched chain amino acids (BCAAs) taken up through the Slimfast transporter can induce up-regulation of TORC1 (Fig. 1) [53–58]. Therefore, we fed flies a slurry of BCAAs diluted 1/3 into standard food (S1 Table), and assessed *Wolbachia* titer in oogenesis. *Wolbachia* titer in the BCAA condition was reduced to 77% of the control (Fig. 4B). This was indicated by an average of 137 +/- 9.71 *Wolbachia* in control oocytes (n = 34) versus 105 +/- 8.48 *Wolbachia* in oocytes from the BCAA condition (n = 33) (p = 0.015) (Fig. 4B). The data suggest that TORC1 stimulation with BCAAs drives oocyte titer reduction, opposite the effects of the TORC1 inhibitor, Rapamycin.

To further investigate a possible role for TORC1, we genetically manipulated a key regulator of TORC1 activity. Tsc2, known as Gigas in *Drosophila*, is downstream of the insulin receptor (Fig. 1) [59–64]. If Tsc2 function is suppressed by any means, this allows TORC1 to become active (Fig. 1) [46,64–68]. Therefore, we tested the impact of *Tsc2* on oocyte *Wolbachia* titer by expressing *Tsc2* dsRNA under the control of germline- and soma-specific *GAL4* drivers [69–72]. This investigation revealed different oocyte *Wolbachia* titer responses to tissue-specific *Tsc2* RNAi knockdowns. Our efforts to manipulate Tsc2 dosage in germline cells had no impact on oocyte titer (Fig. 4C). An average of 182 +/- 13.5 *Wolbachia* were detected in control oocytes (n = 53), which was not significantly different from the 207 +/- 17.7 *Wolbachia* detected in response to germline *Tsc2* RNAi (n = 56) (Fig. 4C). By contrast, *Tsc2* RNAi knockdowns in the somatic cells reduced oocyte *Wolbachia* titer to approximately 50% of the control level (Fig. 4D). Control oocytes exhibited an average of 402 +/- 43.4 *Wolbachia* (n = 24). However, oocytes somatic Tsc2 knockdown flies exhibited an average of 181 +/- 19.8 oocyte *Wolbachia* (n = 21) (p < 0.001) (Fig. 4D). As such, these data implicate somatic Tsc2, and thus somatic TORC1 signaling, in regulation of oocyte *Wolbachia* titer.

### Yeast suppression of oocyte *Wolbachia* titer is mediated by insulin-TORC1 signaling

A role for somatic TORC1 in regulating oocyte *Wolbachia* titer raised the question of whether dietary yeast stimulates TORC1. This could occur through either protein- or insulin-based mechanisms (Fig. 1). As yeast is major source of protein for *D*. *melanogaster*, perhaps its amino acid content stimulates TORC1 to ultimately suppress oocyte *Wolbachia* titer. To test this possibility, we exposed flies to food enriched in Bovine Serum Albumin, prepared specifically to match the protein content of yeast-enriched food (S1 Table). Oocyte *Wolbachia* titer was similar for control and BSA-enriched conditions, however, with the control exhibiting 1260 +/- 102 *Wolbachia* (n = 26), and the BSA-enriched condition exhibiting 1190 +/- 48.2 *Wolbachia* (n = 18) (S2 Fig). This suggests that amino acid availability in the host diet has little impact on oocyte *Wolbachia* titer.

An alternate possibility is that yeast-enriched diets affect oocyte *Wolbachia* through insulin stimulation of TORC1. It was previously shown that dietary yeast stimulates insulin-producing cells (IPCs) the brain to release the insulin-like-peptides (Dilps) into the hemolymph [73,74]. To test whether yeast acts through somatic Dilp secretion to oocyte *Wolbachia* titer, we ablated the IPCs in the brain of fully mature *Drosophila* females. This is achieved using a *dilp2*: *Gene-Switch-GAL4*, *UAS*: *Reaper* system that specifically kills off the brain IPCs in response to a 2-week mifepristone treatment [74].

**Fig 5:**
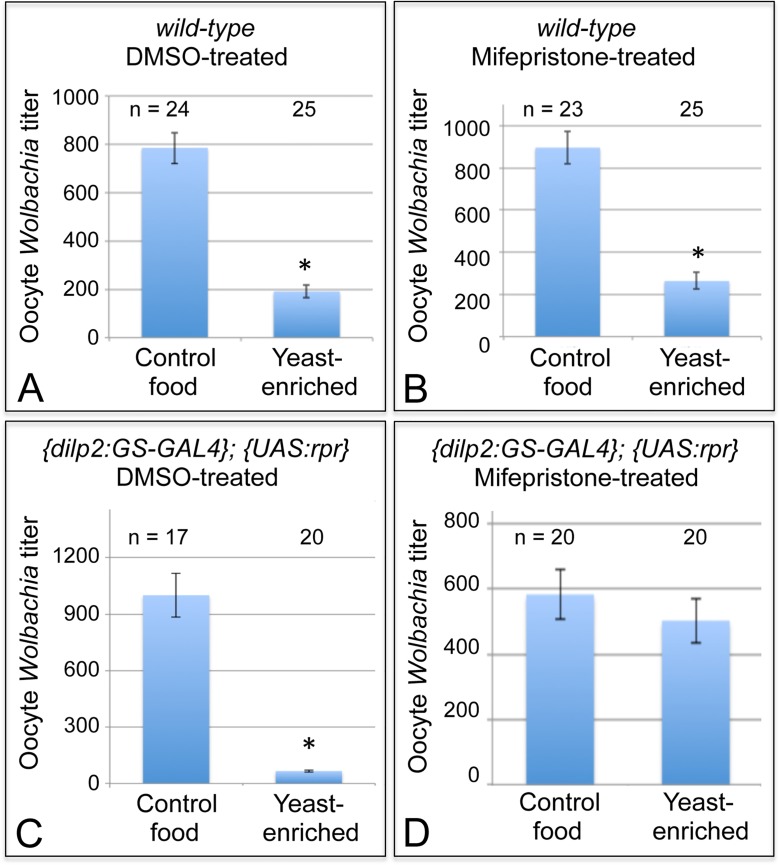
Nutrients affect germline *Wolbachia* titer through the somatic insulin pathway. Dietary impact on oocyte *Wolbachia* titer was tested in flies that either carried or lacked functional IPCs in the brain. Wild-type flies were A) treated with DMSO or B) induced with Mifepristone over a 14-day period as a control. *{dilp2*:*GS-GAL4}; {UAS-rpr}* flies were also C) treated with DMSO as a control, or D) induced with Mifepristone over a 14-day period to drive IPC lethality. * indicates significant changes in titer.

We first investigated whether mifepristone on its own modulates the yeast effect in wild-type flies. After completing a two-week exposure to either DMSO or mifepristone, flies were exposed to either control or yeast-enriched food for 3 days, and their oocyte titer levels were assessed. DMSO-treated flies exhibited substantial oocyte titer depletion in response to yeast-enriched food, down to 30% of the titer in the control condition (Fig. 5A). This was indicated by 785 +/- 64.8 *Wolbachia* per oocyte in the DMSO-control food condition (n = 24), in contrast to 191 +/- 26.9 *Wolbachia* in the DMSO-yeast-enriched condition (n = 25) (p <. 001) (Fig. 5A). Mifepristone-treated flies showed a similar titer reduction after exposure to yeast, exhibiting 21% of the titer seen in the control food condition (Fig. 5B). This was indicated by 896 +/- 77.2 *Wolbachia* per oocyte in the mifepristone-control food condition (n = 23), versus 264 +/- 39.5 *Wolbachia* in the mifepristone-yeast-enriched condition (n = 25) (Fig. 5B) (p <. 001). Therefore, mifepristone alone has no effect on yeast-based suppression of oocyte *Wolbachia* titer.

Next, the exact same treatment regimens were performed on flies with the *dilp2*: *Gene-Switch-GAL4*, *UAS*: *Reaper* genotype. In this experiment, DMSO-treated flies, which retained functional IPCs, exhibited a severe oocyte *Wolbachia* depletion in response to yeast-enriched food, exhibiting only 7% of the oocyte titer seen on DMSO-control food (Fig. 5C). This was indicated by the presence of 999 +/- 116 *Wolbachia* per oocyte in the DMSO-control food condition (n = 17), versus 66.5 +/- 6.61 *Wolbachia* in the DMSO-yeast-enriched condition (n = 20) (p < 0.001) (Fig. 5C). In stark contrast, mifepristone-treated flies that had lost their IPCs exhibited no oocyte titer change after exposure to yeast (Fig. 5D). This was indicated by detection of 583 +/- 72.6 *Wolbachia* per oocyte in the mifepristone-control food condition (n = 20), versus 503 +/- 68.0 *Wolbachia* in the mifepristone-yeast-enriched condition (n = 20) (Fig. 5D). Since mifepristone in combination with the *dilp2*: *Gene-Switch-GAL4*, *UAS*: *Reaper* system specifically prevented yeast from affecting oocyte *Wolbachia* titer, this demonstrates that somatic IPCs mediate *Wolbachia* titer suppression by dietary yeast.

### Dietary sucrose elevates oocyte *Wolbachia* titer in an insulin-dependent manner

**Fig 6:**
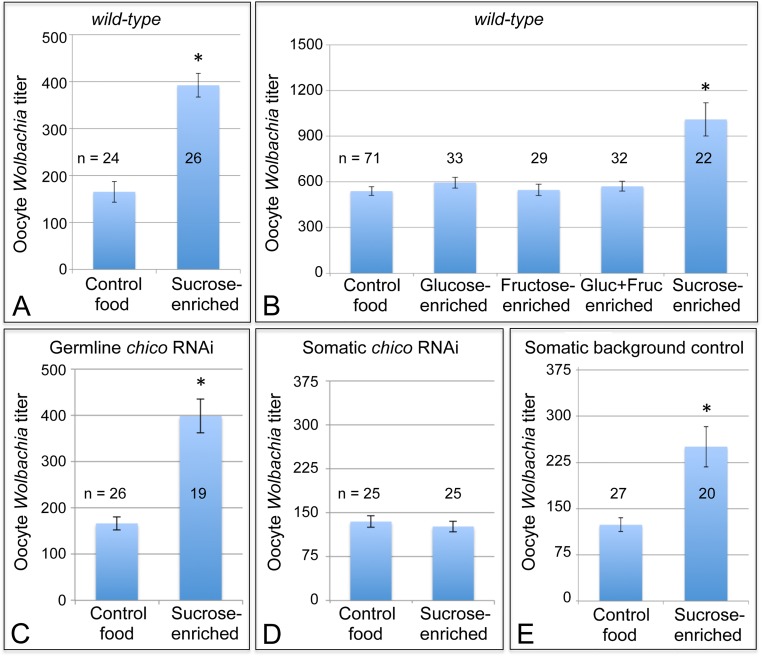
Sucrose-enriched food elevates oocyte *Wolbachia* titer in a *chico*-dependent manner. *Wolbachia* were quantified within single focal planes of oocytes exposed to control food or sucrose-enriched food. The average titer detected per nutrient condition is shown. A) Impact of sucrose on oocyte *Wolbachia* titer in wild-type *D*. *melanogaster*. B) Comparison of oocyte *Wolbachia* titers between control food and other foods enriched in glucose, fructose, a mixture of glucose and fructose, or sucrose. C-E) Sucrose impact on oocyte *Wolbachia* titer in flies that carry tissue-specific *chico* RNAi disruptions. Genotypes used: C) *{nos-GAL4}/+; {nos-GAL4}/{UAS-chico dsRNA}*. D) *{da-GAL4}/{UAS-chico dsRNA}*. E) *{da-GAL4}/+*.

To further investigate the sensitivity of oocyte *Wolbachia* titer to somatic insulin signaling, we also examined the effect of a sucrose-rich, high sugar diet. High sugar diets have been shown to induce insulin resistance in *Drosophila* [75,76]. This is may be due in part to increased expression of NLaz [75], which in mammals is known to suppress Akt function within the insulin signaling pathway (Fig. 1) [77–79]. To test the impact of sucrose-enriched diets on oocyte *Wolbachia* titer, 2-day old *D*. *melanogaster* were fed standard food diluted 1/3 with saturated sucrose solution, hereafter referred to as “sucrose-enriched food” (S1 Table). After 3 days of exposure to this diet, *Wolbachia* titer was assessed in oogenesis. Oocytes from the sucrose-enriched condition exhibited a 2.4-fold increase in *Wolbachia* (Fig. 6A). Unlike oocytes raised on control food, which exhibited an average of 165 +/- 22.2 *Wolbachia* (n = 24), *D*. *melanogaster* oocytes exposed to sucrose-enriched food exhibited 392 +/- 25.3 *Wolbachia* (n = 26) (p < 0.001) (Fig. 6A). These data indicate that a high sugar diet significantly elevates oocyte *Wolbachia* titer, possibly via an insulin-related mechanism.

A sucrose-based impact on oocyte *Wolbachia* titer is surprising, as corn syrup-enriched food did not induce a similar effect (Fig. 2D). Notably, sucrose is a disaccharide, composed of glucose and fructose, whereas corn syrup consists mainly of glucose. To elucidate the basis for sucrose-induced titer effects in oogenesis, food enriched for glucose and fructose were also tested. However, none of the monosaccharide-enriched conditions significantly affected oocyte *Wolbachia* titer (Fig. 6B). Control food yielded an average oocyte titer of 478 +/- 27.6 *Wolbachia* per oocyte (n = 71). Similarly, oocytes in the glucose-enriched condition displayed 520 +/- 31.1 bacteria (n = 33), the fructose-enriched food condition resulted in 478 +/- 33.0 *Wolbachia* (n = 29), and a mixture of glucose + fructose yielded 499 +/- 28.0 *Wolbachia* (n = 32). By contrast, oocytes from the sucrose-enriched condition presented 883 +/- 95.4 *Wolbachia* (n = 22) (p <. 001) (Fig. 6B). This confirms that disaccharide sucrose molecule specifically elicits *Wolbachia* titer increases in oogenesis.

To further test the possibility that insulin signaling mediates sucrose impact on ovarian *Wolbachia* titer, we coupled genetic disruptions of the insulin pathway with sucrose-enriched food. Chico is a *Drosophila* homolog of the Insulin Receptor Substrate that relays signals from the Insulin Receptor to AKT kinase, and thus ultimately TORC1 (Fig. 1) [80,81]. Germline and soma-specific GAL4 drivers were used to drive expression of *chico* dsRNA [69–72], and oocyte *Wolbachia* titer was assayed in control and sucrose-enriched conditions. This test did not indicate any effect of germline *chico* RNAi on sucrose-induced oocyte titer elevation, with sucrose-enriched food corresponding to 2.4-fold higher oocyte titer than the control (Fig. 6C). Germline *chico* RNAi oocytes exhibited 125 +/- 10.6 *Wolbachia* when exposed to regular food (n = 26) as compared to 299 +/- 27.2 *Wolbachia* in response to sucrose-enriched food (n = 19) (p < 0.001) (Fig. 6C). By contrast, somatic *chico* RNAi eliminated sucrose-induced titer effects in oogenesis (Fig. 6D). Oocytes from somatic *chico* RNAi flies exhibited 180 +/- 12.9 *Wolbachia* in the control condition (n = 25), as compared to 169 +/- 12.5 *Wolbachia* per oocyte in the sucrose-enriched condition (n = 25) (Fig. 6D). Analysis of sibling controls further indicated that the genetic background for the somatic *chico* RNAi experiment was not responsible for differential oocyte titer responses to sucrose (Fig. 6E). In flies carrying the somatic *da-GAL4* driver used for this experiment, the sucrose-enriched condition continued to exhibit 2-fold more *Wolbachia* than the control food condition. An average of 124 +/- 11.1 *Wolbachia* were detected in control oocytes (n = 27) as compared to 251 +/- 32.8 *Wolbachia* detected in oocytes from the sucrose-enriched condition (n = 20) (p <. 001) (Fig. 6E). Though the complete mechanistic implications of somatic *chico* disruption remain unclear, these data demonstrate that sucrose acts through somatic insulin signaling to elevate oocyte *Wolbachia* titer.

### Oocyte *Wolbachia* titer responses are independent of ovary productivity

These data raise the fundamental question of why diet-modulated insulin signaling affects *Wolbachia* titer so strongly in germline cells. One possibility is that these titer responses are an indirect result of nutrient-induced changes in ovary size and productivity [76]. Yeast-rich diets and insulin signaling are known to drive formation of larger, more productive ovaries [60,76,80,82–91], while high-sucrose diets have the opposite effect [76–79]. To test the contribution of ovary size and productivity variables on oocyte *Wolbachia* titer, we manipulated ovary productivity by controlling female mating. Mating stimulates ovary development, resulting in a moderately sized, productive ovary. By contrast, virgin females exhibit very large ovaries, filled mainly by mature eggs [92–96]. Oocytes from mated versus virgin females revealed similar oocyte *Wolbachia* titers, however (S3 Fig). The mated condition displayed 449 +/- 27.5 *Wolbachia* per oocyte (n = 26), while the virgin female condition that carried 470 +/- 40.6 *Wolbachia* per oocyte (n = 24) (S3 Fig). These data suggest that ovary size and productivity do not serve as the primary determinants of oocyte *Wolbachia* titer.

### *Wolbachia* nucleoid morphology responds to dietary yeast

To further investigate the effects of host diet on *Wolbachia*, we examined *Wolbachia* nucleoid morphology. Other studies indicate that nucleoid morphology can serve as a proxy indicator of replication-associated changes in cell shape, or stress-induced DNA compaction [97–99]. Multiple, zoomed-in images of *Wolbachia* stained with propidium iodide were projected as a single image, and nucleoid shape was measured. The images indicated that *Wolbachia* nucleoid shape differs between nutrient conditions (S4 Fig). To specifically analyze changes in nucleoid length, 120 nucleoids were selected at random from each treatment condition and their lengths were compared. This analysis indicated that 50% of nucleoids in the control condition exceeded 2 μm in length (S4 Fig). The sucrose-enriched condition was similar, with 53% of nucleoids exceeding 2 μm. In the yeast-enriched condition, however, only 37% of nucleoids exceeded this measure (p <. 05). Thus, yeast-enriched food significantly shortened *Wolbachia* nucleoids. We further determined an elongation index (EI), representing bacterial length divided by width, for the same 120 nucleoids per treatment condition as above. This analysis indicated that 50% of nucleoids measured in the control condition had an EI greater than 2. In the sucrose-enriched condition, only 33% of nucleoids showed an EI greater than 2 (p <. 05). In the yeast-enriched condition, even fewer nucleoids showed this degree of elongation, with only 22% of nucleoids exceeding this EI (p <. 001) (S4 Fig). These data indicate that dietary conditions, and especially exposure to yeast-enriched food, alter *Wolbachia* nucleoid morphology in oogenesis. This is consistent with a bacterial physiological response to host diet.

### *Wolbachia* titers are regulated in a tissue-specific manner

**Fig 7:**
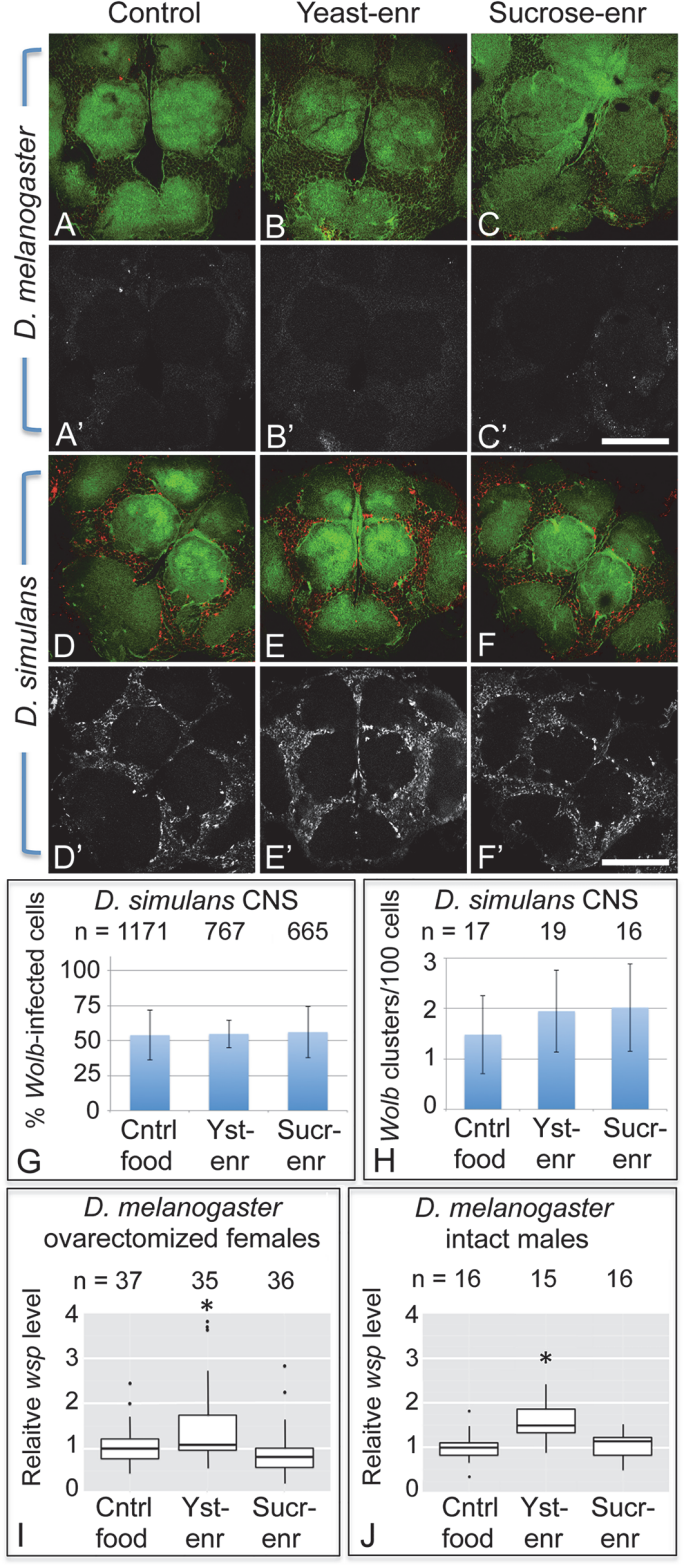
Host diet has tissue-specific effects on somatic *Wolbachia* titer. A-F’) *Wolbachia* in the central brain of female flies. Columns from left to right: Control food, Yeast-enriched, Sucrose-enriched. In merged images, red shows Anti-Wsp to indicate *Wolbachia*, and green shows phalloidin to indicate actin. Grayscale images show only Anti-Wsp. A-C, A’-C’) *D*. *melanogaster* brains. Little Wsp signal is detected under each feeding condition. D-F, D’-F’) Brains from *D*. *simulans*. These show similarly high Wsp immunoreactivity under all feeding conditions. G) Percentage of *Wolbachia*-infected *D*. *simulans* brain cells. H) Frequency of large *Wolbachia* clusters per 100 *D*. *simulans* brain cells. I-J) qPCR analysis of relative *Wolbachia* levels from flies exposed to nutrient-altered diets. The Y-axis shows relative quantitation of genomic *wsp*. Flies used: I) ovarectomized *D*. *melanogaster* females. J) intact *D*. *melanogaster* males. Values are normalized to the control flies in each panel. * indicates a significant change in titer. Scale bars: 150 μm.

The striking impact of dietary nutrients on oocyte *Wolbachia* titer raises the question of whether *Wolbachia* titer in other tissues is responsive to nutrient conditions. *Wolbachia* are present in insect somatic cells, and the *Drosophila* brain is particularly amenable to assessment of somatic *Wolbachia* titer [100,101]. To take advantage of this, we imaged *Wolbachia* in the central brain of *D*. *melanogaster* exposed to different nutrient conditions. This analysis revealed that *D*. *melanogaster* on control food already carry very low *Wolbachia* titer in the central brain (Fig. 7A, A’, n = 3), and flies fed with either yeast-enriched or sucrose-enriched food were indistinguishable in appearance from the control (Fig. 7B, B’, n = 3) (Fig. 7C, C’, n = 3). Thus, *Wolbachia* titer in *D*. *melanogaster* brain does not appear to be affected by the dietary conditions used in this study. An alternative possibility, however, is that the overall low *Wolbachia* titer detected under these conditions hampered our ability to assay nutrient-induced changes in titer.

To pursue this further, the impact of nutrient-altered food was tested in the closely related *D*. *simulans* species, known for carrying high *Wolbachia* titer in its brain cells [101]. Flies exposed to control food exhibited a high titer of *Wolbachia* in the central brain overall (Fig. 7D, D’, n = 7). Similarly high *Wolbachia* titer was detected in the brain after exposure to yeast-and sucrose-enriched food (Fig. 7E, E’, n = 5) (Fig. 7F, F’, n = 4). Further quantification of *Wolbachia* infection frequency did not reveal any differences between nutrient conditions (Fig. 7G). In control food, yeast-enriched, and sucrose-enriched conditions, 55–56% of brain cells exhibited *Wolbachia* infection (n = 1171, 767, and 665 cells, respectively). No differences were seen in formation of large *Wolbachia* aggregates either (Fig. 7H). Brain samples reared on control food, yeast-enriched, and sucrose-enriched conditions all exhibited between 16–19 large bacterial clusters per hundred cells. This indicates that *Wolbachia* titer in the *D*. *simulans* brain is unresponsive to the nutrient-altered conditions used in this study.

To address the possibility that *D*. *simulans* tissues are generally unresponsive to nutrients, we also assessed *D*. *simulans* oocyte titer in response to nutrient-altered food. In contrast to the brain, *D*. *simulans* oocytes exhibited a clear nutrient-dependent *Wolbachia* titer response (S5 Fig). Control oocyte images carried 293 +/- 49.9 *Wolbachia* (n = 10). By contrast, oocyte titer from the yeast-enriched condition was at 40% of the control level, with an average of 116 +/- 20.1 bacteria detected per oocyte (n = 10) (p = 0.004). Furthermore, the sucrose-enriched condition exhibited 2.3-fold higher titer than the control, with 662 +/- 73.6 *Wolbachia* detected per oocyte (n = 10) (p = 0.001) (S5 Fig). Thus, *D*. *simulans Wolbachia* titers are capable of responding similarly to nutrient conditions as *D*. *melanogaster*.

To further probe the impact of host diet on somatic *Wolbachia* titer, we analyzed relative amounts of *Wolbachia* versus host DNA in ovarectomized female flies. In this analysis, females were exposed to nutrient-altered diets, dissected to remove ovarian tissues, and analyzed by qPCR. The results indicate the relative level of *Wolbachia* per host genome copy number. This analysis indicated that yeast-enriched dietary conditions led to higher levels of *Wolbachia* than the control food condition (Fig. 7I). Control samples exhibited a mean relative level of *Wolbachia* of 0.989 (n = 37), whereas the yeast-enriched condition displayed a mean relative level of *Wolbachia* of 1.28 (n = 35) (p < 0.05). Females exposed to sucrose-enriched diets were not significantly different from the control, however, exhibiting a mean *Wolbachia* relative level of 0.792 (n = 36) (Fig. 7I). This titer response profile differs from analyses of *Wolbachia* titer in the ovary as well as the brain. This suggests that host diet affects *Wolbachia* titers in a tissue-specific manner.

As host nutrition has a different impact on ovarian versus somatic *Wolbachia* titers, this raises the question of what would happen in organism lacking ovarian tissue altogether. To address this issue, qPCR analysis was performed on intact male flies. This indicated that bodywide *Wolbachia* titer also increases in response to yeast-enriched food, although not sucrose-enriched food (Fig. 7J). The control food condition carried a mean *Wolbachia* relative level of 1 (n = 16), in contrast to the yeast-enriched condition, which displayed a mean *Wolbachia* relative level of 1.545 (n = 15) (p < 0.05). Sucrose-enriched diets corresponded to a mean *Wolbachia* relative level of 1.027 (n = 16). This analysis confirms that the profile of bodywide titer responses in males is equivalent to ovarectomized females. This suggests that somatic *Wolbachia* titers overall respond to host dietary conditions in a consistent manner.

## Discussion

The major finding of this study is that dietary intake by *Drosophila* strongly influences *Wolbachia* titer in the host female germline: a high yeast diet decreases *Wolbachia* oocyte titer and a high sucrose diet increases *Wolbachia* oocyte titer. This finding adds to a small but growing literature on the impact of host diet on endosymbionts [1,15,16]. Prior studies of *Wolbachia* suggest that this endosymbiont relies heavily upon host provisioning of amino acids and carbohydrates [102–104]. A very recent study analyzing the *Drosophila* midgut and ovary surprisingly indicated that neither dietary yeast nor sucrose had any affect on the *Wolbachia*:host genomic ratio in those tissues [105]. The image-based analyses of this study demonstrate that yeast and sucrose affect germline *Wolbachia* titer at the cellular level, however. It is unclear why *Wolbachia* titer in the oogenesis should be particularly sensitive to diet and whether this is an adaptive response to changes in the host metabolic environment. The evolutionary success of *Wolbachia* depends on its ability to localize at the posterior pole of the oocyte, the site of germline formation. Significantly, we find that *Wolbachia* localize to the posterior pole regardless of whether the host is exposed to the low titer, yeast-enriched diet, or the high titer, sucrose-enriched diet. This suggests the previously described microtubule and motor protein based mechanisms driving posterior localization of *Wolbachia* [38] are robust, even in the face of dramatic titer changes caused by nutrient-altered diets.

**Fig 8:**
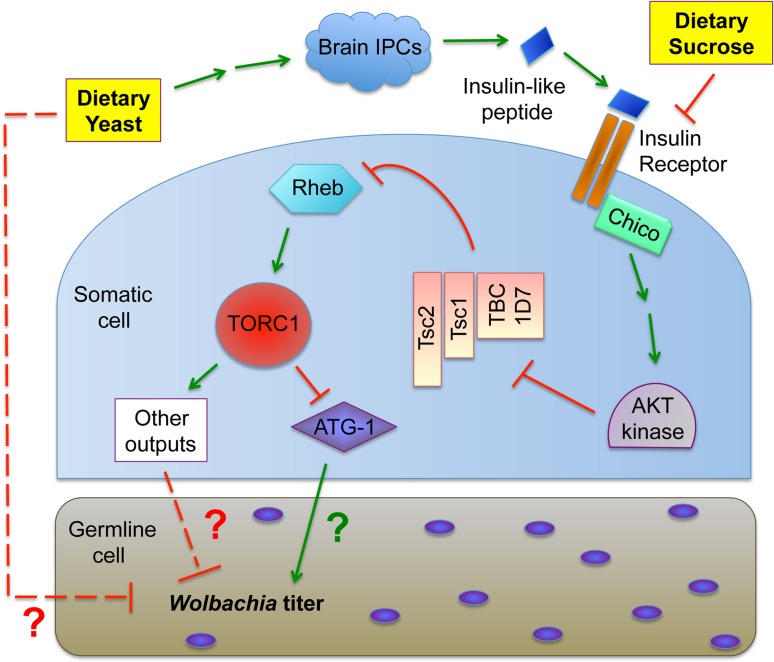
Model for the impact of host diet on germline *Wolbachia* titer.

Insight into the mechanism of yeast-induced titer suppression comes from our functional studies demonstrating that this response is mediated through TORC1. Genetic up-regulation of TORC1 suppresses oocyte *Wolbachia* titer, whereas drug-based inhibition of TORC1 increases titer. This finding creates the basis for a sensible functional connection between intracellular *Wolbachia* and host diet, as both amino acids and insulin signaling are known to drive TORC1 activity [46]. Our finding that BSA-enriched food had no effect on oocyte *Wolbachia* titer argues that yeast protein content is not the major determinant of germline titer suppression, and alternatively suggests a role for insulin signaling. Prior work has shown that yeast-rich diets trigger insulin signaling in *Drosophila*, and that *Wolbachia* interact with host insulin signaling processes [89,106]. Our finding, that loss of somatic IPCs eliminates yeast impact on oocyte *Wolbachia* titer, confirms that insulin signaling facilitates the titer-suppressing effects of yeast. Furthermore, disrupting the somatic insulin receptor substrate, Chico, suppressed the impact of dietary sucrose on oocyte *Wolbachia* titer. This suggests that both dietary yeast and sucrose affect germline *Wolbachia* titer via antagonistic impacts on somatic insulin signaling (Fig. 8).

In considering the mechanism of insulin-based impact on germline *Wolbachia* titer, one possibility is that changes in ovary productivity are responsible. Diet-modulated insulin signaling affects the relative rates of germline stem cell division, germline cell survival and egg chamber development [60,76,80,82–91]. If *Wolbachia* are unresponsive to nutrient-induced adjustments in germline cell growth and development, significant titer changes in oogenesis would be expected. However, oocyte *Wolbachia* titers were very similar in mated and virgin females, despite the different rates of germline stem division expected for each type of flies [76,83,86,88,90–96]. Another possibility is that yeast-induced insulin signaling affects *Wolbachia* physiology in oogenesis. The “rounded” *Wolbachia* nucleoids visible in the yeast-enriched condition could indicate substantially slowed bacterial growth or a bacterial stress response, for example [97–99]. Insulin signaling has been shown to induce changes in cytoskeleton organization, proteasome activity and chaperonin activity [107–111], any of which could affect *Wolbachia* physiology. It is also possible that dietary yeast in particular carries one or more bioreactive agents that are toxic to germline *Wolbachia* (Fig. 8).

The impact of somatic insulin signaling on germline *Wolbachia* titer also raises the question of whether somatic *Wolbachia* titers are similarly affected by host nutrient conditions. Our initial findings that *Wolbachia* titers in the *Drosophila* brain are non-responsive to host diet suggested that nutrient-associated titer changes are restricted to the ovary. Analysis of sucrose-fed, ovarectomized females is further consistent with that interpretation. However, analysis of ovarectomized females also indicated that dietary yeast triggers somatic titer changes opposite of oogenesis. It is possible that this occurs by physical relocation of *Wolbachia* within the body, with dietary yeast driving *Wolbachia* egress from ovarian cells, followed by invasion of somatic target tissues. Alternatively, host dietary conditions may drive tissue-specific differences in the *Wolbachia* life cycle. Perhaps yeast-enriched diets favor *Wolbachia* replication and survival in specific somatic tissues while disfavoring the same in oogenesis. Support for this hypothesis comes from our finding that yeast-enriched food induces the same bodywide titer changes in male flies as seen in ovarectomized females. This demonstrates that ovarian *Wolbachia* titer responses are distinct from that of other tissues.

The pathways downstream and upstream of TORC1 that mediate yeast-based suppression of *Wolbachia* germline titer are yet to be determined. An obvious possibility is the role of TORC1 in suppressing autophagy (Fig. 8). There are numerous examples in which autophagy either enhances or suppresses intracellular bacteria titer [112]. Since TORC1 disruptions increase *Wolbachia* titer in oogenesis, it is possible that *Wolbachia* interact positively with autophagy, consistent with other endosymbionts [113] [114]. As insulin signaling is expected to down-regulate autophagy (Fig. 1), the low *Wolbachia* titers seen in yeast-fed oocytes are further consistent with this possibility. However, the finding that dietary yeast also increases somatic *Wolbachia* titers implies that somatic autophagy is normally bactericidal in that context, consistent with another recent report [115]. These conflicting results may indicate that tissue-specific differences in autophagy regulation contribute to *Wolbachia* titer control, or that other mechanisms downstream or independent from autophagy are responsible (Fig. 8). Perhaps responses from one or more other TORC1 effectors further contribute to *Wolbachia* titer regulation (Fig. 1).

*Wolbachia* have been shown to suppress replication of RNA viruses in insects, including the human pathogens, Dengue Fever Virus and Chikungunya Virus [116–118]. This finding, together with the fact that *Wolbachia*-induced Cytoplasmic Incompatibility rapid spreads *Wolbachia* through insect populations [25,119], has led to a novel strategy of combating these diseases by releasing *Wolbachia*-infected insect carriers of these viruses into afflicted regions [120,121]. Although the mechanism of *Wolbachia*-induced viral suppression is unknown, several studies demonstrate that the higher the *Wolbachia* titer, the greater the viral suppression [122–126]. Our finding that host diet dramatically affects tissue-specific *Wolbachia* titers suggests that the natural diets of the released insects should be taken into account when evaluating the potential effectiveness of a *Wolbachia*-based viral suppression field study. Finally it will be of interest to determine whether diet has a similar effect on *Wolbachia* titer in disease-associated filiarial nematodes.

## Materials and Methods

### Fly strains

Natural *D*. *melanogaster* and *D*. *simulans* flies were harvested daily from collection buckets distributed in the Santa Cruz, CA area. As the female flies of these species are morphologically indistinguishable, but both species were well-represented in the area, this wild-caught population was presumed to represent both species. The laboratory strain of *D*. *simulans* used was a *w-* stock that carried the endogenous *wRi Wolbachia* strain. The *D*. *melanogaster* strain used for the initial nutrient feeds and for crossing *wMel Wolbachia* into the other fly strains was *w; Sp/Cyo; Sb/TM6B*. Other *D*. *melanogaster* fly strains used were the *gigas* VALIUM20 TRiP line: *y*, *sc*, *v; P{TRiP*.*HMS01217}attP2/TM3*, *Sb*; the *chico* VALIUM20 TRiP line: *y*, *sc*, *v; P{TRiP*.*HMS01553}attP2/TM3*, *Sb*; the somatic daughterless driver: *w; P{w+*, *GMR12B08-GAL4}attP2*; the germline triple driver: *P{otu-GAL4*::*VP16*.*1}; P{GAL4-Nos*.*NGT}40; P{GAL4*::*VP16-Nos*.*UTR}MVD1*; and the stocks used for IPC ablation: *w; P{w+*, *dilp2*::*GS-GAL4}/Cyo*, and *w; P{w+*, *UAS*::*Reaper}*. During this work, *wMel* was introduced into the somatic daughterless driver, the germline triple driver, and the *dilp2*::*GS-GAL4* driver, and the infected versions of these stocks were crossed to the *TRiP* or *UAS*:*Reaper* responders. *DrosDel* isogenic flies carrying wMel were used for real-time quantitative PCR analyses [122].

### Food preparation and administration

The standard food recipe used was based upon that of the Bloomington Drosophila Stock Center [127]. The food was prepared in large batches that consisted of 20L water, 337g yeast, 190g soy flour, 1325g yellow corn meal, 96g agar, 1.5L Karo light corn syrup and 94mL propionic acid. To create yeast paste for this study, live bakers yeast was mixed together with water to create a smooth, thick paste. To create the “control food” used in this study, we mixed together 1.5mL ddH2O and 3.5mL of melted standard food in a narrow-mouthed vial, then let cool in an ice bucket to solidify the food suspension. The same procedure applied to creation of all other nutrient-altered foods used in this study. For “corn-syrup-enriched” food condition, 1.5mL Karo light corn syrup was used. For “yeast-enriched” food condition, 1.5mL of heat-killed yeast paste was used. The “BSA-enriched” food carried 0.4g BSA, 1.5mL water, and 3.5mL standard food. For the “sucrose-enriched”, “glucose-enriched” and “fructose-enriched” foods, fresh sugar solutions were prepared at a final concentration of 1g/mL, then 1.5mL of this concentrate was combined with 3.5mL standard food for each vial. The “glucose + fructose enriched” condition carried 0.75mL of 1g/mL glucose, 0.75mL 1g/mL fructose, and 3.5mL standard food. Alternate methods were used to prepare food for the other treatments. For the branched chain amino acid condition, the control condition contained 400μL water and 50μL DMSO mixed with 4.5mL standard food, whereas the experimental condition carried 200μL of 1mg/mL Arginine, 200uL of 1mg/mL Isoleucine and 50μL DMSO mixed with 4.5mL standard food. For the TORC1 testing, 50μL of either control DMSO or 30mM rapamycin/DMSO stock was mixed into 5mL standard food. For tests of IPC function, 50μL of either control DMSO or a 10mM mifepristone-DMSO stock was mixed into 5mL standard food.

Laboratory *Drosophila* stocks were maintained on standard food at 23–24°C. Identical population density was used in all vials, and control and experimental conditions run in parallel. Flies of the genotype *w; Sp/Cyo; Sb/TM6B* were used in all imaging experiments that assessed nutrition as the only variable. In the cases where crosses were needed to drive expression from *TRIP* line stocks or the *dilp2*:*GAL4* stocks were used, we performed all crosses using identical population density and female age distribution in all vials, with control crosses always run in parallel. Virgin female flies were collected during the first 3 days of eclosion only, then subjected to nutrient conditions. The procedure was to collect a range of 0–24 hour old adults, age these young flies for 2 days on standard food, and expose to treatment conditions for 3 more days. The mixture of *D*. *melanogaster* and *D*. *simulans* flies collected from nature likely varied in age. These flies were also exposed to standard food for 2 days, and transferred to experimental food for 3 days. In the case of IPC ablation, the collected flies were allowed to mature 2 days, then transferred to mifepristone-containing food or DMSO control food. The flies were maintained on this food for 14 days, transferring the population to a fresh vial every 3 days of the treatment period. After this was completed, the flies were exposed to nutrient-altered food for 3 days.

### Tissue staining, imaging, and analysis

Samples were prepared from a minimum of 10–15 flies per condition in each replicate. Ovary dissection, fixation, and propidium iodide staining were done as previously described in order to label germline *Wolbachia* nucleoids [38]. Ovarian tissues for all samples in each replicate were mounted on slides in parallel to ensure maximal consistency in sample compression between slide and coverslip. All samples were then imaged on a Leica SP2 confocal microscope at 63X magnification with 1.5X zoom. Experimental samples verified to exhibit the same degree of compression as the control sample were pursued further, while any experimental samples deviating from that were discarded. Z-series images were acquired from each egg chamber of interest at 1.5 μm intervals. Uniform intensity settings were applied to all egg chambers imaged within each replicate. A minimum of 7–10 oocytes were ultimately imaged from each condition, with all experimental oocytes matched for morphological consistency against control oocytes of the same replicate. Using this rigorous method, significant fold-differences in *Wolbachia* titer were consistently identified between control and experimental conditions, regardless of the baseline quantity of *Wolbachia* detected in each replicate.

To quantify *Wolbachia* titer in the confocal images, we used established methods to identify the deepest possible focal plane where *Wolbachia* are clearly visible in all samples tested for each replicate [32]. The images were processed in Photoshop to remove everything from the images except oocyte *Wolbachia*, which were then quantified using the Analyze Particles feature in Image J. This analysis ultimately quantifies the *Wolbachia* nucleoids carried per oocyte, or per nurse cell, within a single, representative focal plane of each egg chamber. Although the graphical data displayed in the figures present all experimental averages as normalized against the control averages, all statistical calculations were run by comparing each condition only against controls that were run in parallel. Significant differences were indicated by ANOVA. A minimum of 2–3 replicates were performed for most germline staining experiments described in this study. The only exception was the experiment in which *Wolbachia* titer responses were analyzed in both brain and ovary tissues. In that case, single replicates were done for each type of tissue stained, with all conditions run in parallel.

To analyze *Wolbachia* titer by real-time quantitative PCR, single flies were homogenized with a pestle in 250 μl of Tris HCl 0.1M, EDTA 0.1M and SDS 1% (pH 9) and incubated for 30 minutes at 70 ºC. After 35 μl of KAc were added the sample was incubated 30 minutes on ice, centrifuged for 15 minutes at 13.000 rpm at 4ºC and the supernatant stored. Samples were diluted 100x for qPCR. qPCr was performed as described previously [122], using the CFX384 Real-Time PCR Detection System and iQ SYBR Green Supermix (both BioRad). The relative amount of *Wolbachia* was calculated with the Pfaffl method [128], using the primers for the gene *wsp* to determine *Wolbachia* DNA levels and primers for host *Rpl32 and Actin5C* genes to normalize male and female samples, respectively [122]. Data from males were analyzed using a linear model on the log of the relative *wsp* levels (Im in R) [129]. Data from females were analyzed using a mixed linear model on the logs of relative *wsp* levels (lmer in R).

To analyze *Wolbachia* in the *Drosophila* central nervous system, brains were dissected and fixed as previously described [101]. Brains were incubated in anti-rabbit wsp antibody + PBST (0.1% Triton X-100) for 4 hours at room temperature or at least 12 hours at 4 degrees. For secondary antibody staining, goat anti-rabbit Alexa Fluor 546 (Invitrogen) was used at room temperature or at least 12 hours at four degrees. Actin labeling was done with phalloidin conjugated to Alexa 488, diluted 1:100 in PBST, for one hour at room temperature. Brain tissues were imaged on a Leica SP2 confocal microscope at 63X magnification. Brains were quantified with Leica LAF AS software. One representative focal plane per brain was scored. Cells containing one or more *Wolbachia* were scored as infected. *Wolbachia* aggregates larger than 10 microns^2^ were scored as a “cluster” [101].

To assess *Wolbachia* nucleoid shape, we acquired Z-series images of stage 10A oocytes at 63X magnification with 5X zoom. Then we created a projection of 4 images from each Z-series, located just beneath the follicle cell layer, and measured the length of individual nucleoids using the “line” tool located within the Profile function of Quantification Tools in the Leica SP2 software. Elongation index was calculated as a function of length divided by width. It is assumed that the bacteria are random in orientation, and thus detecting a range of nucleoid morphologies ranging from spherical to rod-shaped is possible. Chi square tests were used to compare *Wolbachia* length and elongation index exhibited by bacterial populations from each treatment condition.

## Supporting Information

S1 Table Nutritional content of the food types administered. This table displays combined information from the USDA National Nutrient Database for Standard Reference, Release 27, scaled to the volumes of ingredients used for each condition. The protein content of the branched chain amino acid (BCAA)-enriched food, noted with an asterisk, represents the combined weight of the added amino acids plus other protein present in the food. The nutritional content of glucose-enriched, fructose-enriched, and glucose+fructose enriched food were nearly identical to sucrose-enriched food according to the nutrient classifications used in this table, and thus are not shown.
(TIF)

S1 Fig Host diet affects oocyte *Wolbachia* titer in wild-caught *Drosophila*. *Wolbachia* nucleoids were quantified in the oocytes of wild-caught *D*. *melanogaster* and *D*. *simulans*. Control and yeast-enriched feeding conditions were used. * indicates a significant change in titer.
(TIF)

S2 Fig BSA-enriched food has no impact on oocyte *Wolbachia* titer. Female *D*. *melanogaster* were exposed in parallel to control and BSA-enriched food conditions, and their *Wolbachia* nucleoids were quantified in oogenesis. Average titer levels are shown.
(TIF)

S3 Fig Oocyte *Wolbachia* titer is unaffected by mating. Oocyte *Wolbachia* nucleoids were quantified in *D*. *melanogaster* females that had either been reared together with males or maintained in isolation from males. Average titer levels are shown.
(TIF)

S4 Fig Dietary conditions affect *Wolbachia* nucleoid morphology. A-C) Zoomed-in views of *Wolbachia* nucleoids in *D*. *melanogaster* oocytes. Treatments: A) Control fly food. B) Yeast-enriched food. C) Sucrose-enriched food. D) Assessment of *Wolbachia* nucleoid length in response to nutrient conditions. E) Quantification of elongation index exhibited by the same bacteria. * indicates a significant change in titer. Scale bar: 10 μm.
(TIF)

S5 Fig Nutrient-altered food affects oocyte *Wolbachia* titer in *D*. *simulans*. The *D*. *simulans* flies used for this preparation were raised, exposed to nutrient-altered food, and stained in parallel with the *D*. *simulans* analyzed in Fig. 6 A-F’. * indicates a significant change in titer.
(TIF)
