# Correction: The Impact of Host Diet on *Wolbachia* Titer in *Drosophila*

**DOI:** 10.1371/journal.ppat.1004889

**Published:** 2015-05-11

**Authors:** Laura R. Serbus, Pamela M. White, Jessica Pintado Silva, Amanda Rabe, Luis Teixeira, Roger Albertson, William Sullivan

The following information is missing from the Funding section: LT received funding from the Fundação para a Ciência e Tecnologia, PTDC/SAU-MII/105655/2008.
