# Supplementary figures and images for: The Impact of Host Diet on Wolbachia Titer in Drosophila
Source: PLoS Pathog. 2015 Mar 31;11(3):e1004777. doi: 10.1371/journal.ppat.1004777 (PMC4380406; doi:10.1371/journal.ppat.1004777)

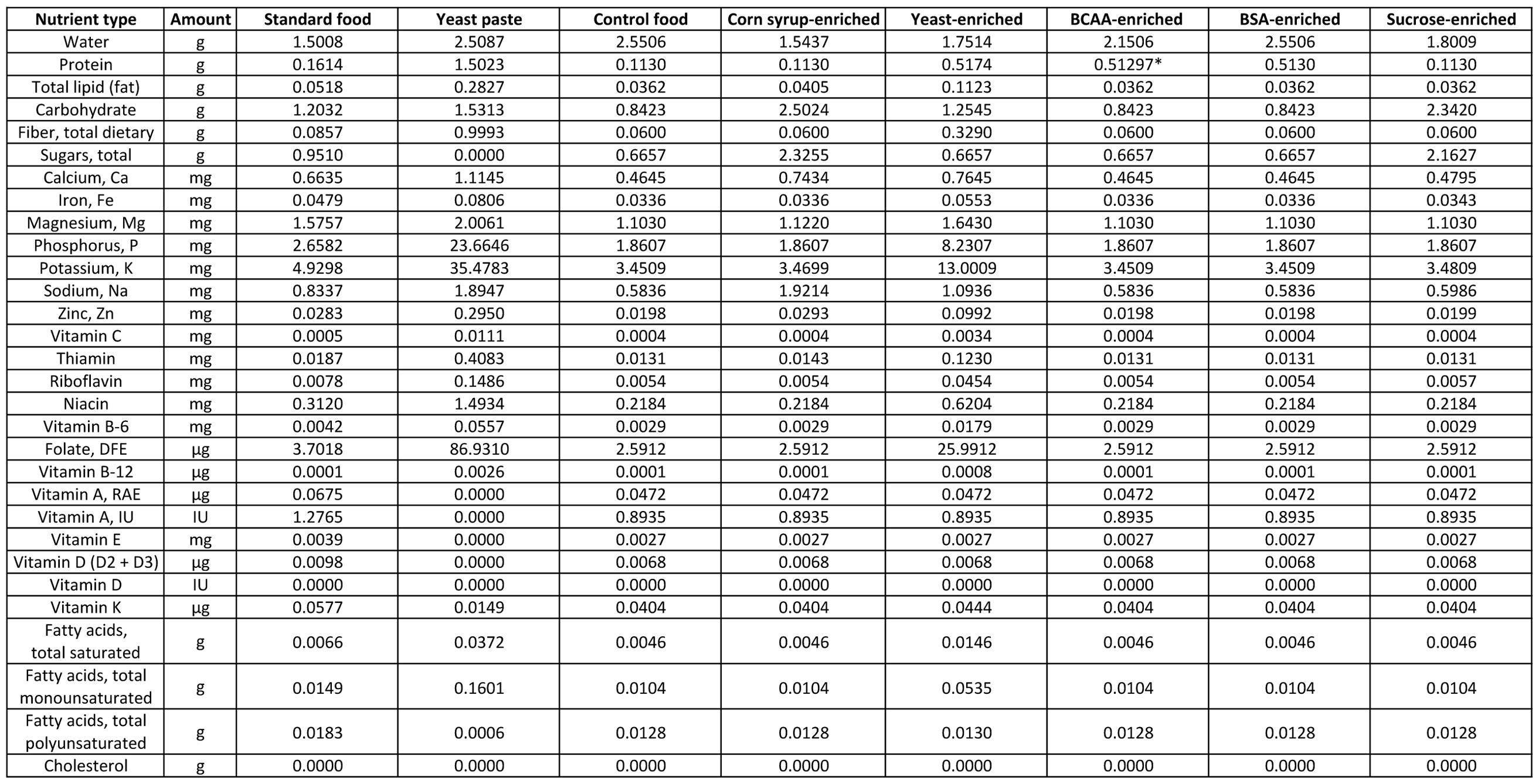

Supplement: S1 Table — This table displays combined information from the USDA National Nutrient Database for Standard Reference, Release 27, scaled to the volumes of ingredients used for each condition. The protein content of the branched chain amino acid (BCAA)-enriched food, noted with an asterisk, represents the combined weight of the added amino acids plus other protein present in the food. The nutritional content of glucose-enriched, fructose-enriched, and glucose+fructose enriched food were nearly identical to sucrose-enriched food according to the nutrient classifications used in this table, and thus are not shown. (TIF) [file ppat.1004777.s001.tif]

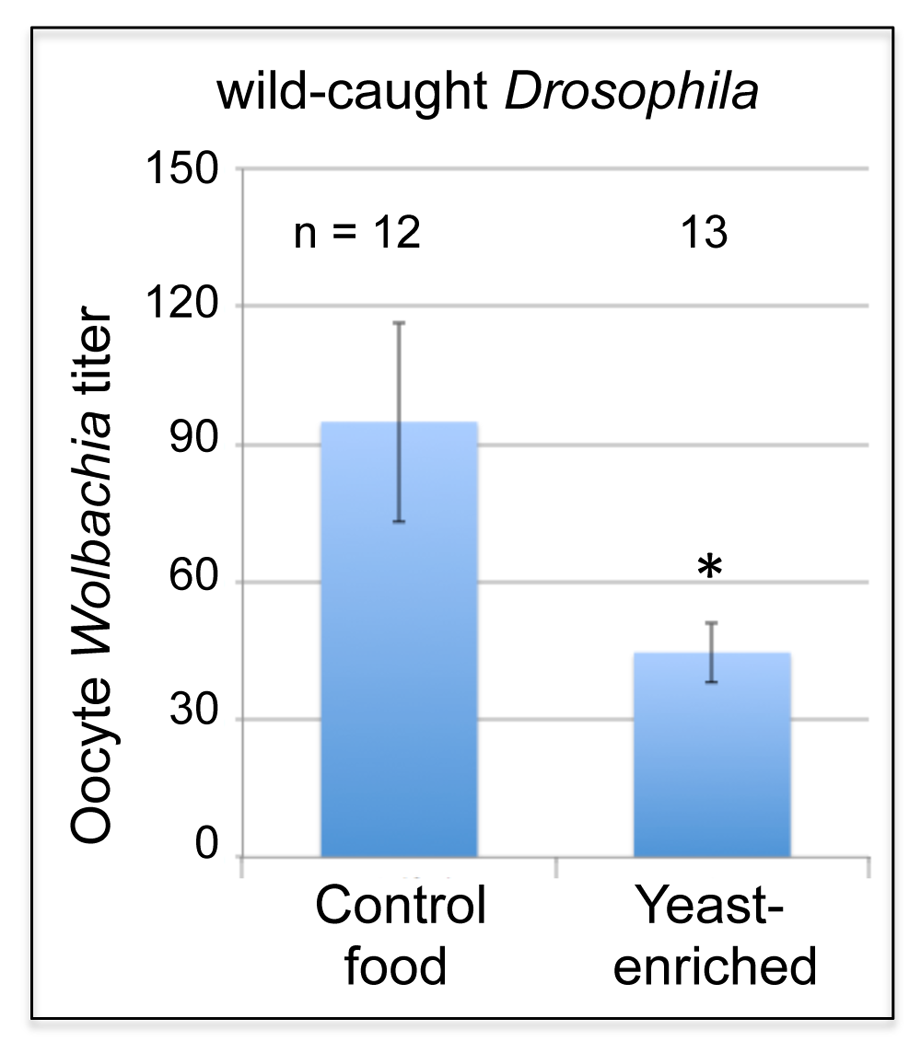

Supplement: S1 Fig — Wolbachia nucleoids were quantified in the oocytes of wild-caught D. melanogaster and D. simulans. Control and yeast-enriched feeding conditions were used. * indicates a significant change in titer. (TIF) [file ppat.1004777.s002.tif]

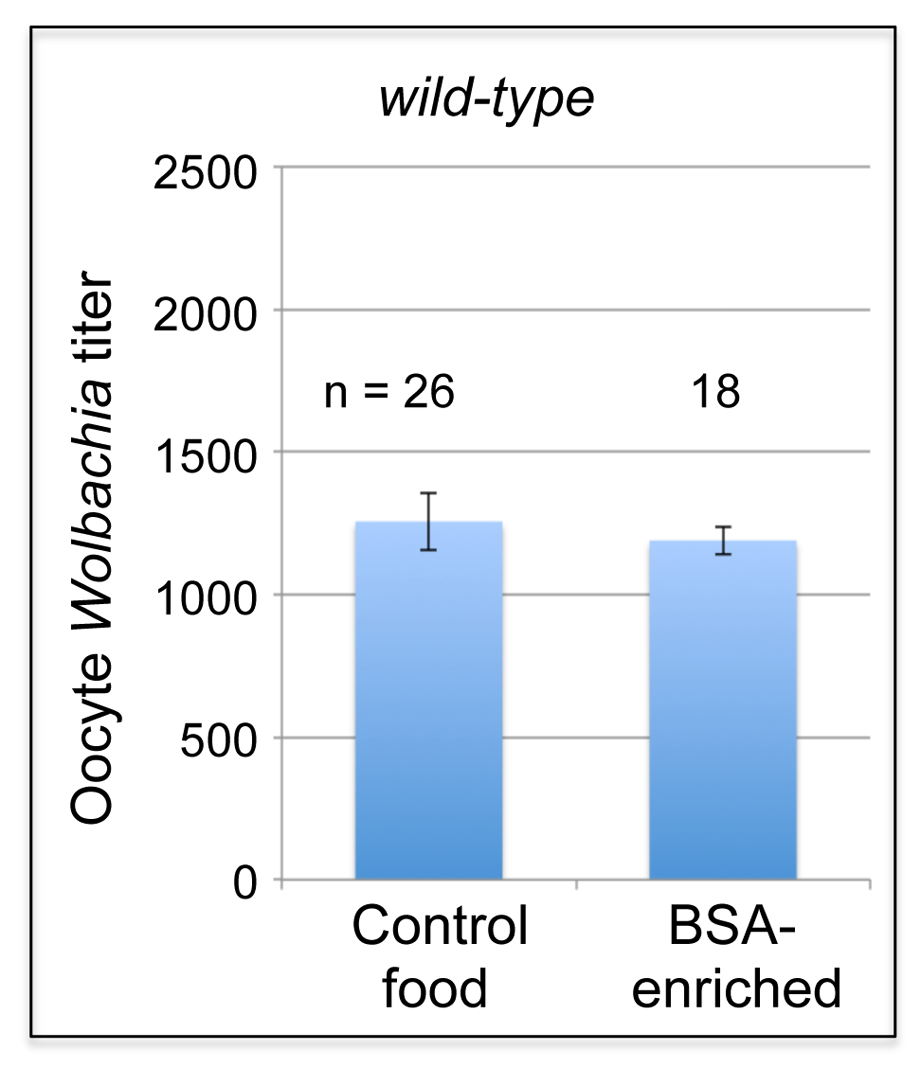

Supplement: S2 Fig — Female D. melanogaster were exposed in parallel to control and BSA-enriched food conditions, and their Wolbachia nucleoids were quantified in oogenesis. Average titer levels are shown. (TIF) [file ppat.1004777.s003.tif]

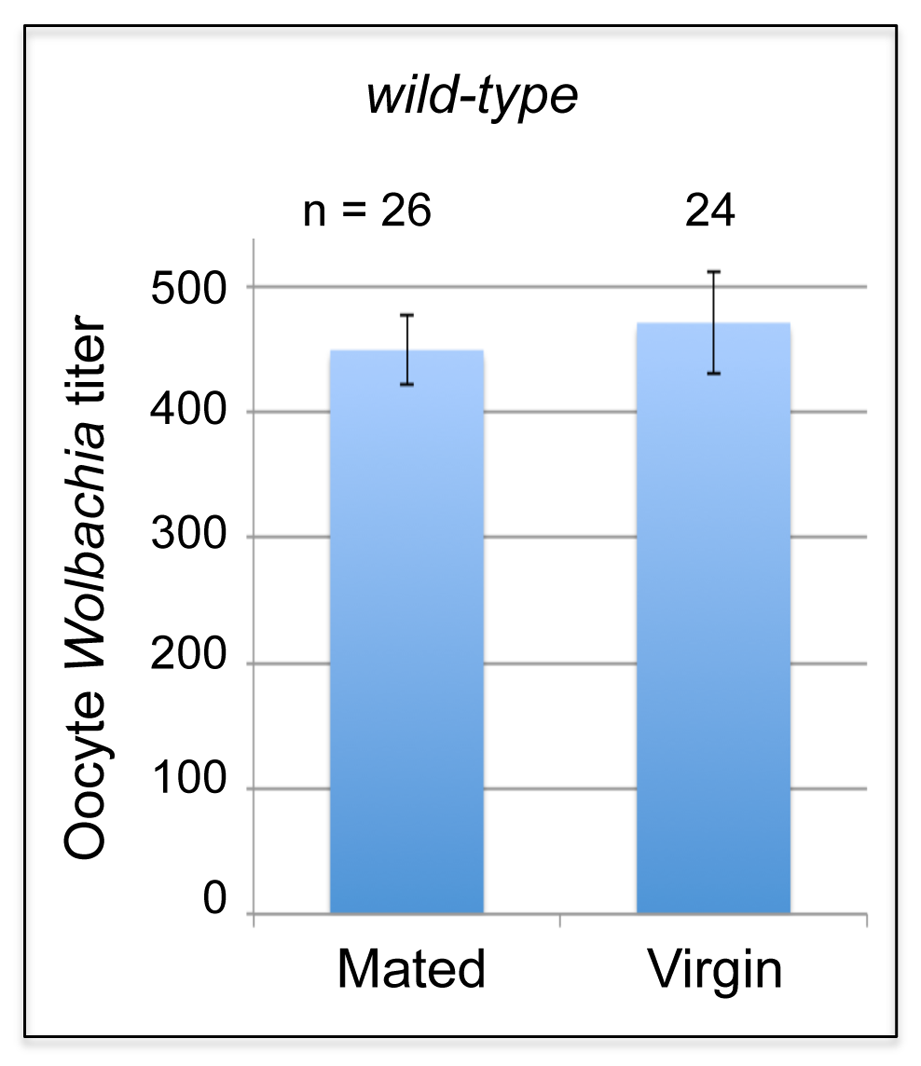

Supplement: S3 Fig — Oocyte Wolbachia nucleoids were quantified in D. melanogaster females that had either been reared together with males or maintained in isolation from males. Average titer levels are shown. (TIF) [file ppat.1004777.s004.tif]

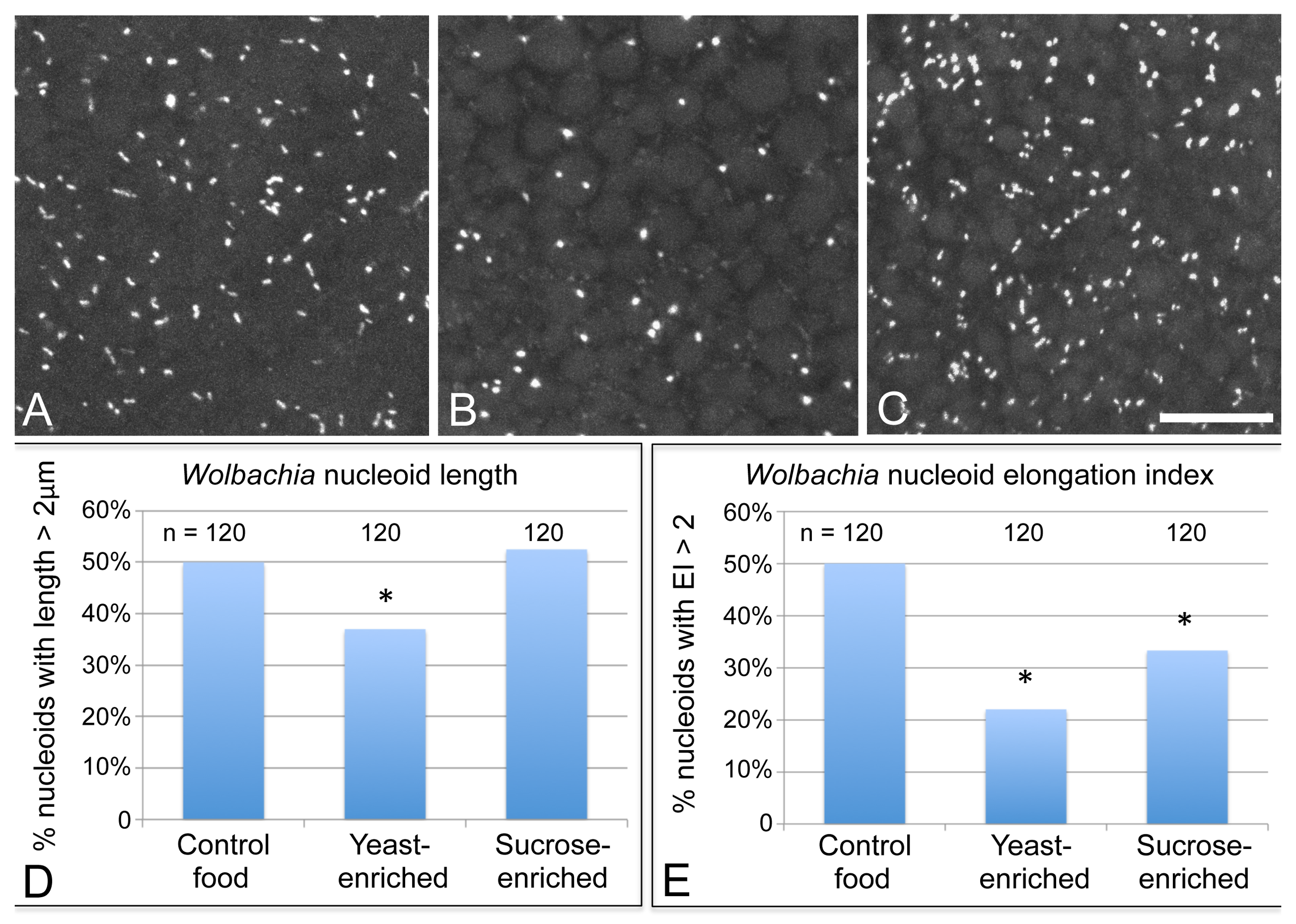

Supplement: S4 Fig — A-C) Zoomed-in views of Wolbachia nucleoids in D. melanogaster oocytes. Treatments: A) Control fly food. B) Yeast-enriched food. C) Sucrose-enriched food. D) Assessment of Wolbachia nucleoid length in response to nutrient conditions. E) Quantification of elongation index exhibited by the same bacteria. * indicates a significant change in titer. Scale bar: 10 μm. (TIF) [file ppat.1004777.s005.tif]

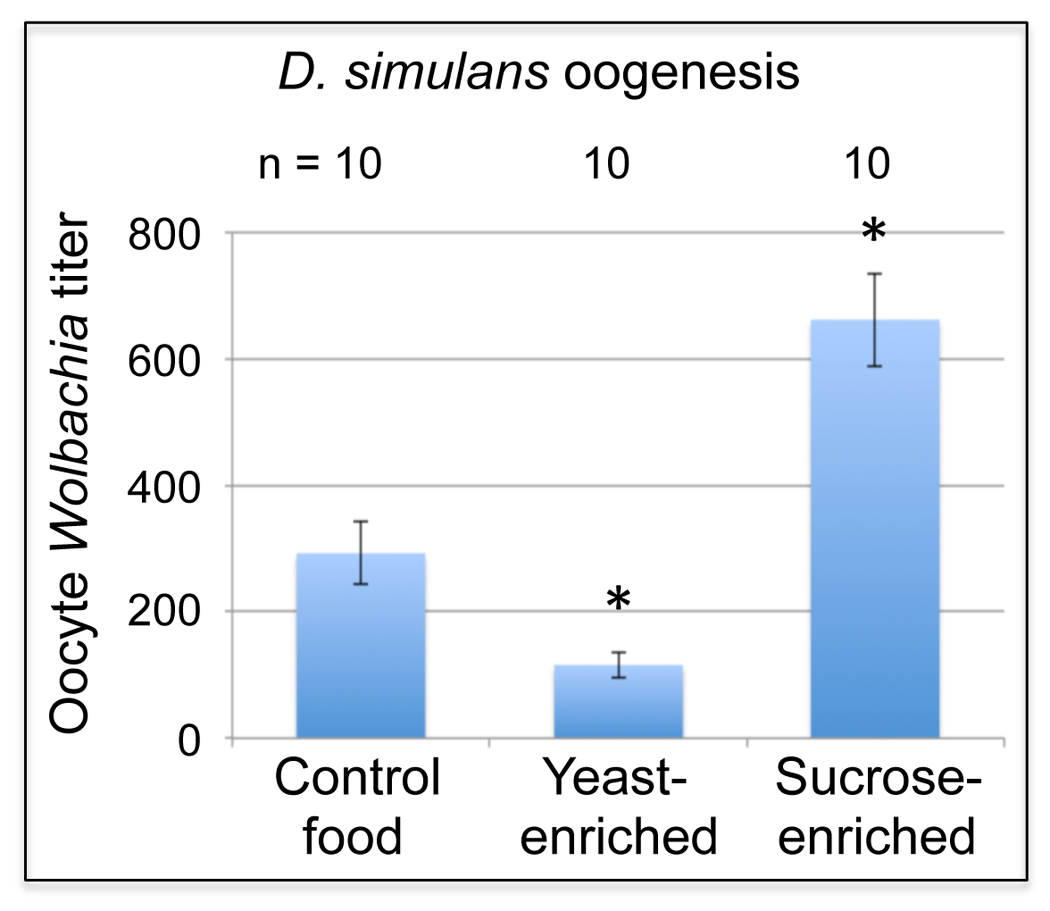

Supplement: S5 Fig — The D. simulans flies used for this preparation were raised, exposed to nutrient-altered food, and stained in parallel with the D. simulans analyzed in Fig. 6 A-F’. * indicates a significant change in titer. (TIF) [file ppat.1004777.s006.tif]
